# Mid-term results of previously cemented hip arthroplasties revised with uncemented modular femoral components: a retrospective study

**DOI:** 10.1186/s13018-015-0266-9

**Published:** 2015-08-14

**Authors:** Tahir Mutlu Duymus, Zafer Solak, Yusuf Ozturkmen, Ibrahim Azboy, Serhat Mutlu, Mustafa Caniklioglu

**Affiliations:** Department of Orthopaedics, Kanuni Sultan Suleyman Training and Research Hospital, Istanbul, Turkey; Department of Orthopaedics, Medical Park Hospital, Ordu, Turkey; Department of Orthopaedics, Istanbul Training and Research Hospital, Istanbul, Turkey; Department of Orthopaedics, Dicle University Medical School, Istanbul, Turkey; Orthopedics and Traumatology Department, Kanuni Sultan Suleyman Education and Research Hospital, Atakent Mahallesi, Turgut Ozal Caddesi,No:1, 34303 Kucukcekmece Istanbul, Turkey

**Keywords:** Revision hip arthroplasty, Uncemented modular stem, Femoral stability, Vertical subsidence, Cortical index

## Abstract

**Background:**

We evaluated the mid-term results of previously cemented hip arthroplasties revised with uncemented modular femoral components.

**Methods:**

The study included 40 patients (36 females (90 %) and 4 males (10 %), mean age 67.6 years, range 39–87 years) who underwent revision of a previously cemented hip prosthesis with an uncemented modular femoral stem between 2005 and 2009. The indications for revision were femoral aseptic loosening in 38 (95 %) cases and acetabular protrusion in 2 (5 %). According to the Paprosky classification, the femoral defect was type 1 in 10 (25 %) patients, type 2 in 16 (40 %), type 3a in 11 (27.5 %), type 3b in 2 (5 %) and type 4 in 1 (2.5 %). The Harris hip score was used for the clinical evaluation. Femoral vertical subsidence, the cortical index and femoral stem stability were assessed radiologically. The mean follow-up period was 84 months (range 61–95 months).

**Results:**

The mean Harris hip score was 41.4 (range 35.4–44.4) preoperatively and 80.9 (range 65.6–98.3) at the final follow-up examination (*p* < 0.05). Mean vertical subsidence was 5.7 mm (range 2.5–10.5 mm) in seven (17.5 %) patients. Stable bone fixation was observed in 38 (95 %) patients, fibrous stable fixation in 2 (5 %) and no instability in any patient. Radiographs taken during the early postoperative period revealed that the cortical index was a mean of 1.34 (range 1.11–1.73) and a mean of 1.55 on the final follow-up radiographic examinations (range 1.16–1.91) (*p* < 0.01).

**Conclusions:**

Satisfactory results were achieved using uncemented modular femoral components during revision of previously cemented femoral components. Many modular femoral stems provide primary stability by filling femoral bone losses and help determine stem length, offset and anteversion.

## Background

The number of hip prosthesis revision surgeries has increased due to the increasing numbers of primary hip prosthesis applications. Revision surgery is costly, and selecting the type of femoral components for revision arthroplasty is controversial. Due to the poor results of cemented femoral components after revision surgery, uncemented and modular stems have become more popular [1, 2]. The advantages of modular uncemented stems include reduced stress shielding by filling the proximal and distal femur optimally and providing better primary stability and providing easier offset and anteversion. The disadvantages are the increased risk of intraoperative fracture in the metaphyseal-diaphyseal area during implantation and irreversible bone loss in the bone stock of young patients, in particular, which can result in later re-revision difficulties [3].

We conducted a retrospective evaluation of mid-term radiological and functional results in patients who underwent revision surgery with uncemented modular femoral components due to aseptic femoral loosening that developed following primary cemented hip arthroplasty.

## Methods

This study included 40 patients (36 females (90 %) and 4 males (10 %), mean age 67.6 years, range 39–87 years) who underwent primary cemented hip arthroplasty and revision with uncemented modular femoral stems (Helios; Biomet, Valencia, Spain) between 2005 and 2009. The indications for primary arthroplasty were osteoarthritis in 8 (20 %), hip fracture in 21 (52 %), coxarthrosis based on developmental hip dysplasia in 9 (22 %), coxarthrosis following hip tuberculosis in 1 (3 %) and femoral head avascular necrosis in 1 (3 %). Partial hip arthroplasty was applied to 12 of 21 patients because of hip fracture, and total hip arthroplasty was applied to 9. Revision surgery was applied to the right hip in 12 (30 %) cases and to the left hip in 28 (70 %). The indications for revision were femoral aseptic loosening in 38 (95 %) cases and acetabular protrusion in 2 (5 %). The acetabular component was changed in 24 (86 %) due to loosening of the acetabulum. An acetabular ring was used in one patient, and a jumbo cup was used in two.

According to the Paprosky classification, the femoral defect was type 1 in 10 (25 %) patients, type 2 in 16 (40 %), type 3a in 11 (27.5 %), type 3b in 2 (5 %) and type 4 in 1 (2.5 %). The mean follow-up period was 84 months (range 61–95 months).

**Fig. 1:**
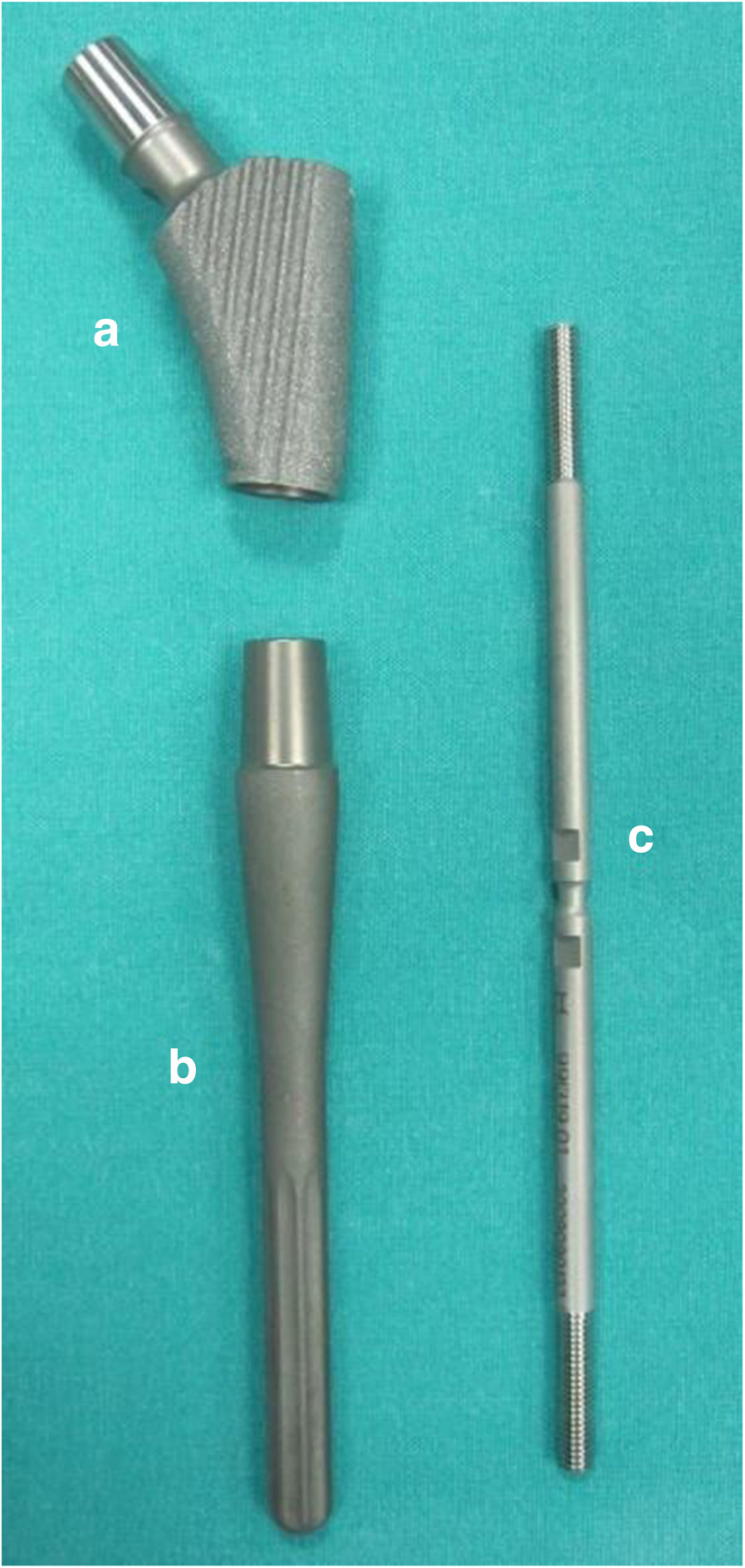
HELIOS Modular Type Revision System components. **a** Proximal (metaphyseal part): 40, 50, 60, 70 and 80 mm. **b** Distal part: diameters of 10, 12, 14, 16 and 18 mm; lengths of 120, 160, 200 and 240 mm. **c** Locking screw (one size)

An uncemented modular femoral stem was used in all patients. The prosthesis was composed of a titanium-aluminium-niobium compound, and the metaphyseal component surface was roughened with a porous coating. This system consisted of the three uncemented components. The shaft of the modular-type revision prosthesis was smooth with a 145° neck angle. Eight lengthening edges were on the distal end of the prosthesis. The proximal (metaphyseal) piece started at 40 mm and had five alternatives in 10 mm increments. The distal (diaphyseal) piece started with a 10 mm diameter and had five alternatives in 2 mm increments. The four length choices were in the range of 120–240 mm. An intermediate locking part was placed between the proximal and distal parts (Fig. 1).

Pelvic, hip, and anteroposterior (AP) and lateral femoral radiographs were taken of all patients for preoperative planning. Femoral bone loss was evaluated in all patients according to the Paprosky classification prior to revision surgery [4].

### Surgical technique

**Fig. 2:**
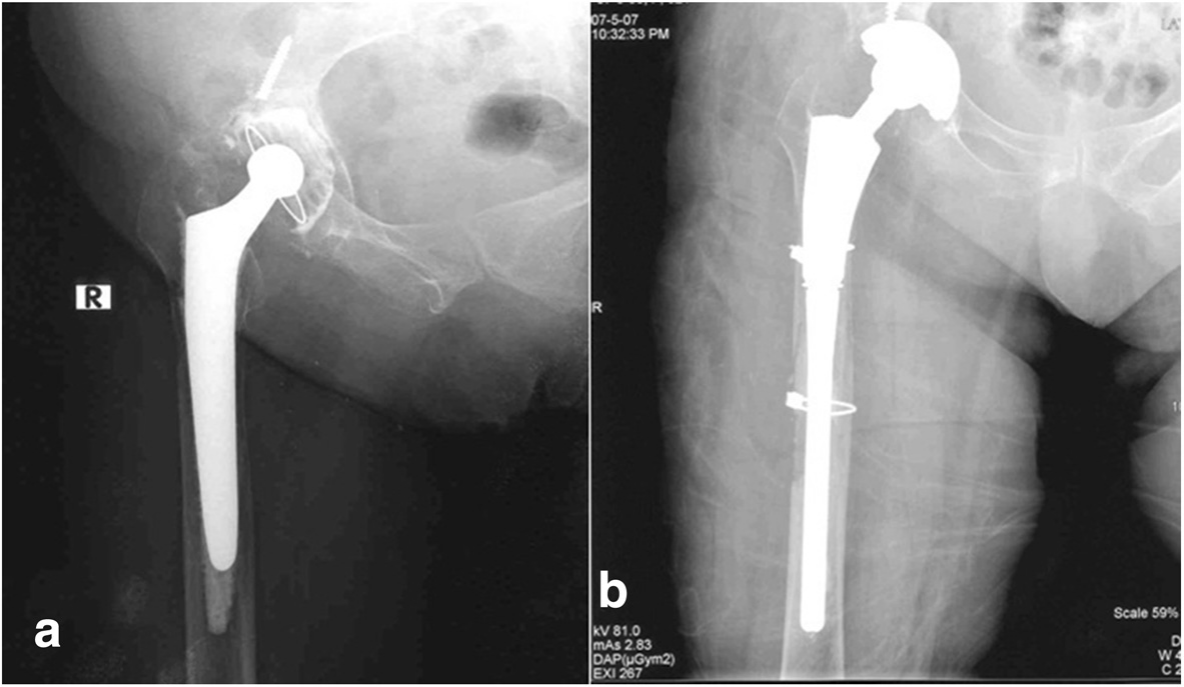
**a** A 62-year-old female underwent cemented total hip arthroplasty for coxarthrosis secondary to a previous operation for an acetabular fracture. Radiograph shows loosening of the femoral stem and acetabular cup. **b** Postoperative radiograph of the revised components. The trochanteric osteotomy flap was sufficiently long to allow good access to the medullary canal to remove the cement and to press-fit implant the stem perfectly

**Fig. 3:**
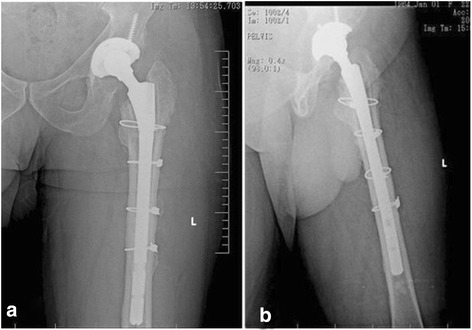
**a** Anteroposterior radiograph. **b** Lateral radiograph of a revised cemented femoral stem demonstrates good fill and fit of the cementless modular stem. The trochanteric osteotomy flap was fixed carefully, and the osteotomy site was consolidated

All patients were placed in the lateral decubitus position. The hip was reached through an extended posterior incision. A window was opened to the femoral diaphysis in four patients, and a 20 % extended trochanteric osteotomy was used in eight patients to completely remove the cement. Then, the osteotomy flap was re-adapted using cerclage wire or a grip cable system. No gap was left between the osteotomy flap and the distal femur to prevent dislocation due to proximalisation of the greater trochanter and the decreased strength of the abductor muscles (Figs. 2 and 3). In addition, subtrochanteric cerclage was applied in all patients to protect against femoral fracture when preparing the femoral groove or implanting an uncemented modular stem. The femur was prepared with hard and flexible reamers. First, the thickness of the diaphyseal component was defined, and a proximal part large enough to fill the metaphyseal defect was selected. A trial reduction was made after placing the acetabular component. Anteversion, stability, limb-length discrepancy, osteotomy reduction and the need for structural allograft were evaluated at this stage.

A structural allograft was used in seven (17.5 %) patients. A grip cable system or cerclage wire was used to fix the trochanteric osteotomy and the structural allograft. A hemovac drain was placed in position when range of movement and stability were sufficient, and the layers were closed.

Cefazolin sodium (1 g) was administered intravenously as antibiotic prophylaxis 1 h before making the initial skin incision and was continued for 24 h at a dose rate of 3 × 1 g. A single daily dose of 40 mg enoxaparin sodium was administered subcutaneously as a thromboemboli prophylaxis starting 12 h preoperatively and was continued for 1 month.

The patients were mobilised with partial weight-bearing under guidance of a physiotherapist on postoperative day 2, and full weight-bearing was permitted within 6 weeks. The patients were evaluated with hip radiographs and standard follow-up procedures at 6 weeks, 3 months, 6 months and 1 year postoperatively.

The Harris hip score (HHS) was used to clinically evaluate hip function preoperatively and at the final follow-up examination [5].

### Radiographic evaluation

Femoral vertical subsidence, cortical index and femoral stem stability were evaluated. Subsidence of the femoral stem was measured by taking fixed points on the femoral bone and stem as references. Stem subsidence was measured by comparing two AP radiographs taken early after surgery and at the final follow-up examination. Thus, the centre of the trochanter minor was accepted as the femoral bone landmark, and the distal edge of the component was considered the reference on the stem. The distance between the middle of the lesser trochanter and the rim of the stem was calculated based on the two X-rays, and the difference between the two represented subsidence in millimetres. Subsidence ≥5 mm was accepted as vertical subsidence [6].

Stability of the femoral stem was evaluated in accordance with the criteria described by Engh et al. [7]:

1. Stable bone fixation, no subsidence of the implant, no radiolucent line around the stem or very little radiolucence.
2. Stable fibrous fixation, no advanced migration. Early mild migration can occur but no extensive radiolucent line around the stem is visible. No local hypertrophy findings in the femoral cortex.
3. Unstable implant. Progressive migration of the stem within the femoral canal. Divergently wide radiolucent lines, at least partially, around the stem. Increased cortical density and thickening immediately below the stem neck and at the end.

The cortical index was evaluated by measuring the ratio of the external diameter of the femur to the width of the medullar canal 1 cm distal to the trochanter minor [8].

Approval for this study was granted by the Local Ethics Committee, and written informed consent was obtained from all participants.

Pre and postoperative HHS values were compared using SPSS 15.0 software (SPSS Inc., Chicago, IL, USA). The Kolmogorov–Smirnov test was used to assess normality of the data distribution. The Wilcoxon test was applied for pre- and postoperative comparisons. A *p* < 0.05 was considered to indicate significance.

## Results

The mean HHS was 41.4 (range 35.4–44.4) preoperatively and 80.9 (range 65.6–98.3) at the final follow-up examination (*p* < 0.05). In addition, the clinical results were not significantly different between the partial hip arthroplasty group (*n* = 12) and the total hip arthroplasty group (*n* = 28). The HHS values at the final follow-up were 80.9 (range 66–96) in the partial hip arthroplasty group and 81.7 (range 68–99) in the total hip arthroplasty group. The postoperative results based on the HHS were excellent in 9 (22.5 %) patients, good in 11 (27.5 %), moderate in 11 (27.5 %) and poor in 9 (22.5 %). Poor results were observed in patients with reduced bone density and more extensive bone loss (Paprosky type III or IV femoral bone loss).

**Fig. 4:**
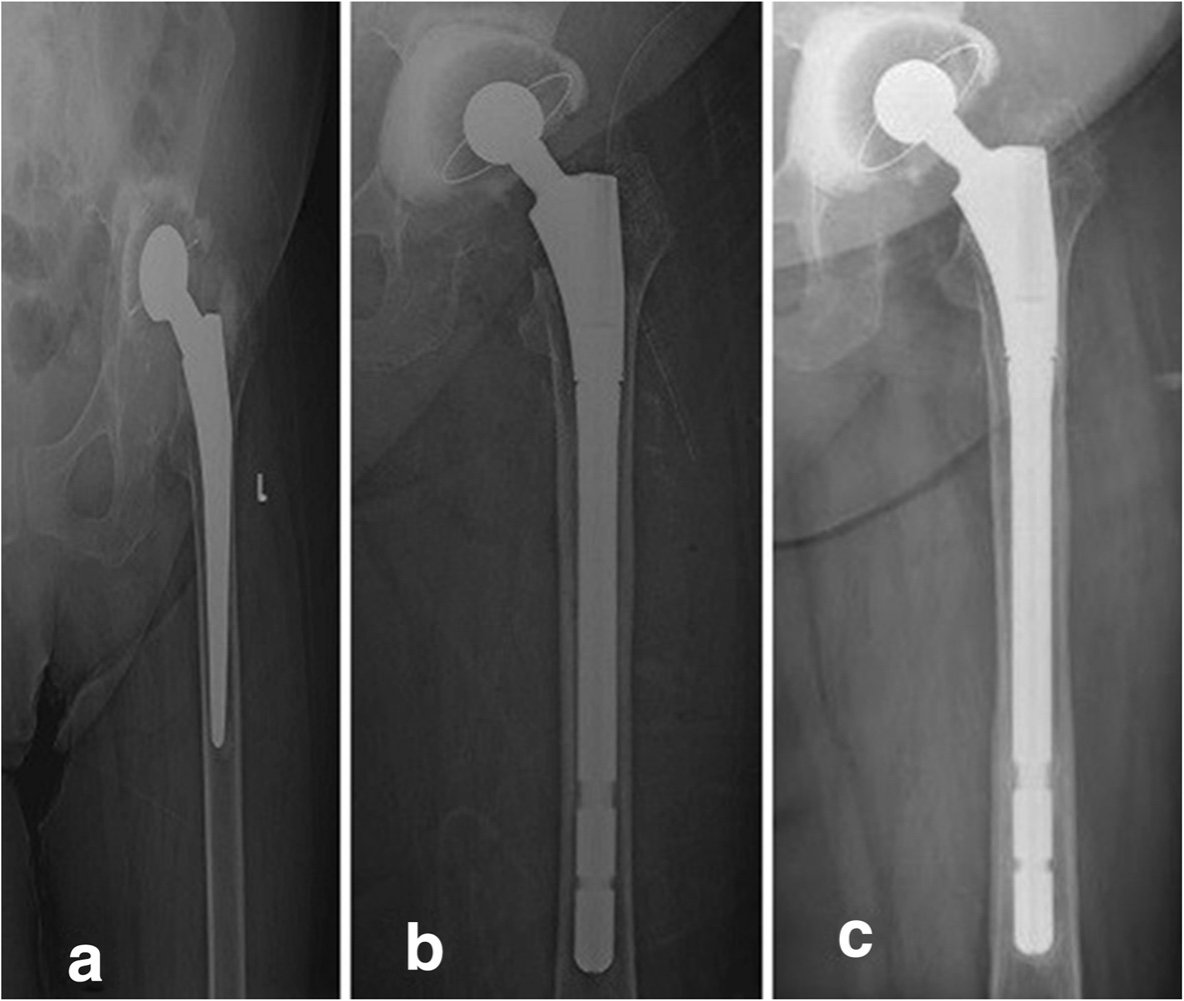
**a** Aseptic loosening of a cemented hip prosthesis implanted 18 years previously in a 68-year-old female. **b** Anteroposterior (AP) radiograph of the hip during the early postoperative period after revision with a modular type proximal porous-coated diaphyseal auto-locking femoral stem. **c** AP radiograph of the hip 72 months after revision

When the immediate postoperative radiographs of the femoral component were compared with those from the final follow-up examination, mean vertical subsidence was 5.7 mm (range 2.5–10.5 mm) in seven patients and >5 mm in four (10 %), in whom vertical subsidence was seen but was not as progressive, and there was no need for revision. Subsidence <5 mm was seen in three patients. Stable femoral stem bone fixation was observed in 38 (95 %) patients, fibrous stable fixation in 2 (5 %) and no instability in any patient. No correlation was found between subsidence and the length or diameter of the femoral component. Mean cortical index was 1.34 (range 1.11–1.73) on radiographs taken immediately after surgery and 1.55 on the final follow-up examination (range 1.16–1.91) (*p* < 0.01). A structural allograft was used in seven hips (17.5 %), and full union with bone was achieved (Fig. 4). Bony consolidation of the trochanteric osteotomy flap was achieved in 38 (93 %) patients on final follow-up radiographs. Fibrous fixation of the flap was observed in the other three (7 %) patients.

No fatal complications developed in any patient during or after surgery. An intraoperative femoral fracture was detected in two patients. Fixation was achieved with cerclage wire, and no problems occurred in either patient during the follow-up. Bursitis developed in one patient due to cerclage wire applied for fixation and recovered with medical treatment. A superficial infection developed postoperatively in two patients, and both responded to antibiotic therapy. Vertical subsidence >5 mm was seen in four hips. No re-revision was required, as this subsidence did not progress during follow-up. No dislocations associated with femoral stem subsidence were observed but dislocations occurred in three patients. One dislocation was attributed to poor cup positioning, which was revised, and the other two dislocations were treated nonoperatively (one resulted from a fall and no clear cause for dislocation was identified in the other). Symptoms developed in the peroneal branch of the sciatic nerve in one patient, as the patient could not dorsiflex the foot, so an ankle-foot orthosis was applied. According to the Broker classification, heterotopic ossification was seen in five type I hips (12.5 %) and two type II hips.

## Discussion

Proper implant selection is required for revision hip arthroplasty, but fixation methods remain controversial. Extensive bone loss around the femoral component and large bone defects, particularly in the proximal metaphysis, are often encountered following aseptic loosening. Proximal femoral bone is lost in stems that can only be fixed distally because of stress shielding and osteolysis [9]. Therefore, it is extremely important to fill both the proximal and distal femoral gaps to provide optimal primary stability. Thus, use of modular femoral components has come to the fore in revision hip arthroplasty surgery.

Due to great variations in metaphyseal and diaphyseal bone loss during revision surgery, it is extremely difficult to obtain proper positioning for length, anteversion and stem offset while placing the stem. It is easier to estimate the stability of the stem and fix it during surgery using a modular femoral stem composed of several parts. Modular femoral stems fill the femoral bone optimally and provide maximal primary stability due to the stem length, thickness, offset and anteversion choices available. Thus, a correct and stable femoral head centre can be obtained [10, 11].

It is often difficult to provide sufficient contact between the implant and bone during revision surgery because of proximal bone loss, and results of proximal porous-coated monoblock stems are unsatisfactory [12, 13].

Extensive defects can be seen, particularly in the proximal femur, due to aseptic loosening when applying a primary cemented hip prosthesis [4]. In the current study, loss of proximal femoral bone before the revision surgery was similar to that reported previously [14]. The most important causes for these defects are osteolysis and loss of cancellous bone detected when removing the cement [15]. A radiological evaluation of all bone stock is important, particularly for periprosthetic bone regeneration. Regeneration of severely altered bone stock is often random soon after a revision. In this situation, additional bone tissue or substitutes are useful [16]. Allografts are preferred to cover extensive bone defects and stabilise the prosthesis [17, 18]. We used allografts to cover defects and stabilise the prosthesis in seven patients. No problems related to incorporating an allograft were observed in those patients.

A relationship has been found previously between femoral stem length, quantity of bone stock and bone density. It has been reported that thinning of the femoral bone and reduced density reduces the bone stock when using a long stem for diaphyseal fixation; therefore, primary fixation from the proximal end and a short stem are recommended [16, 19, 20]. However, despite using a long stem, we found no thinning femoral bone, which may have been associated with the lower number of cases compared to other studies.

No significant relationship was observed in bone stock quantity preoperatively and migration because a modular stem fills the femoral bone optimally both proximally and distally, which increases primary fixation strength. Girard et al. reported that the risk of secondary subsidence does not increase even if the preoperative bone defect is significant [21]. We found no correlation between subsidence and dislocation in our case series. However, none of our cases had subsidence >10.5 mm. Significant secondary subsidence is often due to technical errors during surgery. The area for stem fixation should be prepared carefully to achieve a good fill and fit of a diaphyseal stem [21].

Regeneration of proximal femoral bone is associated with the amount of bone lost in the hip, but evaluating bone regeneration radiographically is difficult. Changes in the femoral cortex and external diameter are often observed on radiographs taken during the early postoperative period [22]. Bohm et al. found an 87 % increase in the cortical index in a monoblock series [23]. Douglas et al. reported a mean increase of 4.1 % in the cortical index in a retrospective study of a 70-patient modular series at a mean 47-month follow-up [24]. In the current study, the cortical index at the final follow-up increased significantly by a rate of 15 %, which is low compared to that reported for monoblock stems series but higher than similar modular-type femoral revisions. The importance of the large difference between these results is unclear. However, patients with a low cortical index may have differences in the ability to restore bone or the fracture-recovery response in those who have undergone an extended trochanteric osteotomy. The cortical index would provide more meaningful results after revision surgery by examining homogenous patients groups and separating those who have undergone extended trochanteric osteotomy from those who have not.

Migration is a significant problem following revision surgery and may be associated with technical errors during surgery or different femoral stem designs [21]. The isthmus region of the femoral stem should be filled to at least 4–5 cm to prevent vertical migration during surgery [25, 26]. Subsidence >5 mm was determined in 34 % of patients, and subsidence >10 mm was observed in 20 % of patients in a study using the Böhm and Bischel monoblock femoral stem. Del Alamo et al. reported a similar high rate of 20 % of patients with subsidence >10 mm [22, 23]. However, only 15 and 20 % of patients had subsidence >5 mm in two studies that examined patients undergoing revision with an uncemented modular type stem [21, 27]. In the current study, subsidence >5 mm was detected in four (10 %) patients.

Mean subsidence in our series was significantly less compared with that in patients who received monoblock femoral stems during the revision. The lack of progressive subsidence in the current study was due to the ability to completely and tightly fill the canal in the metaphysis and distal diaphysis of the component. Even if migration is not fully and absolutely prevented during a revision with a modular stem, it is thought to occur at a lower rate compared to migration in monoblock stems. Slight migration is not serious [28]. Girard et al. reported a negative correlation between osteointegration quality and subsidence; osteointegration quality was very good or good in 24 (83 %) of 29 patients with migration >5 mm [21]. Limited migration increases the contact area between the stem and the femoral cortex; thus, increasing the quality of the osteointegration. As modular stems allow less migration than monoblock stems, they should be used for revision surgery.

The HHS is widely used to evaluate long-term clinical results. The mean increase in the HHS observed preoperatively to postoperatively in the current study was consistent with the literature [29, 30].

Several studies have reported intraoperative femoral fractured using different implant designs and approaches. Some studies have described complications during uncemented femoral stem revisions [29–31]. Intraoperative fractures or fissures occurred at a rate of 24 of 76 cases in a similar study by Pattyn et al. This high rate was not due to placement of a modular stem but because of removal of a primary cemented prosthesis. They concluded that these types of complications would decrease with more surgical experience [32]. The rate of intraoperative femoral fracture associated with stem placement was 8.8 % in a study by Paprosky et al. [26]. A high rate of risk for intraoperative femoral fracture exists when uncemented femoral stems are used, bone quality deteriorates and complication rates increase when stem length and diameter are increased during revision surgery [33]. We found intraoperative fractures caused by implanting a modular femoral stem in two patients (2.5 %). Our low rate of femoral fractures was due to the application of subtrochanteric cerclage prior to implanting the modular stem.

The strength of this study was that it included only cases of aseptic loosening from primary cemented hip arthroplasty. The limitations are that it was a retrospective study, the number of patients was relatively low and it included several types of femoral defect. A clearer conclusion could be reached in studies involving more homogenous defect subgroups.

## Conclusions

We have shown successful and satisfactory mid-term clinical and radiological results using uncemented modular prostheses for hip prosthesis revision surgery. Many modular femoral stems provide good primary stability by filling in the femoral bone loss and facilitated determining stem length, offset and anteversion.
